# Crisis conformity as affiliation defense under mortality salience: a TMT perspective

**DOI:** 10.3389/fpsyg.2025.1597502

**Published:** 2025-07-24

**Authors:** Kexin Lu, Yang Gao, Yichen Ni, Hong Li

**Affiliations:** ^1^Mental Health Education and Counseling Center, Tongji University, Shanghai, China; ^2^School of Public Management, Northwest University, Xi’an, China; ^3^Department of Senior High School, Shenzhen Foreign Language School, Shenzhen, China; ^4^Department of Psychology, Tsinghua University, Beijing, China

**Keywords:** crisis conformity, need for affiliation, mortality salience, affiliation defense mechanism, terror management theory

## Abstract

**Background:**

During crises, individuals often exhibit elevated conformity, as evidenced by crowding, stampedes, and hoarding; however, the mechanisms driving this behavior remain underexplored. As Terror Management Theory suggests, behaviors that intensify after mortality salience serve as existential defenses. Drawing on TMT, this study posits that crisis-induced conformity arises as a defense against death anxiety. Coupled with evidence that conformity fulfills the fundamental need for affiliation, this research hypothesizes that conformity specifically functions as an affiliation defense through which individuals mitigate death-related anxiety in life-threatening contexts.

**Method:**

Four experiments were conducted to test the proposed three hypotheses. Study 1a employed scenario-based priming (crisis vs. daily situations) and self-report measures to investigate the elevated affiliation needs and conformity as affiliation defenses at two levels, in line with the mortality salience hypothesis. Study 1b used an improved TMT paradigm, replacing neutral controls with economic crisis manipulations to isolate existential threats from nonspecific emotional arousal. Study 2 elucidates the relationship between affiliation needs and conformity propensities in crisis contexts by investigating how experimental manipulation of affiliation influences conformity. Study 3 used a 2 (situation: crisis/control) × 2 (conformity/non-conformity) design to investigate the anxiety-buffering function of conformity.

**Results:**

Both Study 1a and 1b showed that crises significantly increased both affiliation needs and the tendency toward conformity. Study 2 revealed that the presence of dyadic companions during a crisis significantly attenuates individuals’ affiliation needs toward crowds, while conformity propensities also significantly diminish. Study 3 found that crisis conformity reduced negative affects and increased positive emotions compared to non-conformity, while no such effects emerged in daily contexts.

**Conclusion:**

These findings reveal the psychological mechanism of crisis conformity within the framework of TMT: crises increase affiliation needs and conformity (Studies 1a & 1b), consistent with the mortality salience hypothesis. The presence of evacuation companions during mortality-salient crises significantly attenuates affiliative needs toward crowds and correspondingly reduces conformity propensities (Study 2). Conformity as an affiliation defense mitigates negative affect and mortality anxiety while enhancing positive affect, thereby buffering psychological distress (Study 3). This study extends TMT to the field of crisis conformity research, providing a new perspective on interpreting the intrinsic motivation underlying crisis conformity.

## 1 Introduction

Conformity is a common yet perplexing phenomenon during crisis events. Similar to daily conformity, crisis-induced conformity may not fulfill the expected goals and can even trigger serious secondary disasters (e.g., stampedes, looting). However, when a crisis strikes, people still instinctively choose to follow the crowd (Helbing et al., 2000; Gao and Li, 2015). The question of why people conform during crises has not received sufficient attention and exploration. People often assume that crisis-related conformity is a reflexive reaction (Gao and Li, 2015). This study aims to explore the underlying psychological processes of crisis conformity.

A crisis refers to a low-probability event characterized by unclear information regarding its cause, outcome, and problem-solving methods; it poses a significant threat to the survival of individuals, groups, or organizations and requires a response within a short period (Li and Gao, 2013; Li et al., 2015). It essentially encompasses natural disasters, public health events, social events, and terrorist attacks (Yang and Zheng, 2009; Zhan et al., 2016). In this research, we focus specifically on existential crisis contexts—situations involving acute threats to physical survival (e.g., fires, earthquakes). A crisis is a series of environmental changes triggered by a crisis event, characterized by high risk, uncertainty, and a threat to life (Sayegh et al., 2004). Compared with the objectively occurring crisis events themselves, which are beyond human control, psychological research focuses more on individuals’ psychological and behavioral responses during crises (Fan et al., 2003; Xie and Zhu, 2011). In this study, we operationalize “crisis” (referring to objective crisis events) and “crisis” (emphasizing event-contingent contexts) as the contextual antecedents of individual psychological shifts under real-world crisis conditions. Conformity is a common response of individuals during crises.

Regarding the reasons for crisis conformity, some studies suggest that conformity may serve as a heuristic strategy during crises (Gigerenzer, 2008; Li and Gao, 2013), enabling individuals to make quick judgments and decisions. However, in terms of actual consequences, conformity may lead to tragic results (Helbing et al., 2000), which is inconsistent with the ecological rationality typically associated with heuristic strategies. Some studies have explored the influencing factors and conditions of crisis conformity and found that the way information is presented (Chen and Li, 2014), the source of information (Li et al., 2015), and the complexity of the decision-making situation (Li et al., 2016) can all affect whether individuals adopt a conformity strategy during crisis evacuation. Similarly, these studies are also insufficient to explain the underlying reasons for crisis conformity: what motivates individuals’ propensity for conformity in crisis contexts? This paper argues that there may be deeper reasons why conformity is immediately activated in a crisis as a heuristic strategy.

Currently, some scholars have attempted to explain the mechanism of the conformity phenomenon during crises. For example, Li et al. (2011) identified key drivers of crisis conformity (e.g., interest stakes, ambiguity, and time pressure) through simulation modeling, while Duo et al. (2016) proposed a fire-conformity model that emphasizes fear, decisional uncertainty, and option availability. However, these models do not address why crises, together with these key factors, evoke conformity despite its risks, necessitating an exploration of motivational drivers that prompt intuitive conformity despite its potential downsides. Therefore, this paper endeavors to analyze this phenomenon from the perspective of Terror Management Theory.

A hallmark of life-threatening crises is existential risk, which aligns with Terror Management Theory’s concept of “mortality salience”—crises act as primes that elevate death awareness (Hayes et al., 2010; Lu et al., 2017). TMT posits that people use a series of psychological defenses to alleviate and cope with the death awareness and existential anxiety triggered by mortality salience. The theory identifies two criteria for verifying whether a psychological phenomenon or behavioral manifestation serves as a defense against death reminders (Hayes et al., 2010; Lu et al., 2017): (1) According to the *mortality salience hypothesis*, mortality salience increases the psychological phenomenon or behavior; (2) according to the *anxiety-buffering hypothesis*, strengthening this phenomenon or behavior can reduce the negative impact of mortality salience on individuals. Based on these assumptions, numerous specific behaviors have been proven to be reinforced, serving as a defense against mortality salience.

The *dual-process model* of TMT delineates how defense operates at distinct stages. When death-related thoughts temporarily enter consciousness, *proximal defenses* pseudo-logically suppress existential vulnerability (e.g., through health-protective behaviors); conversely, when mortality salience activates subliminally, *distal defenses* symbolically transcend human fragility by affirming *symbolic* immortality (Kosloff and Greenberg, 2009). Notably, substantial TMT research has prioritized distal mechanisms—operationalized through post-distractor assessments of defenses presented as unrelated to mortality primes. Three core pathways or mechanisms are established that underlie diverse defensive manifestations: cultural worldviews, self-esteem, and maintaining close relationships.

### 1.1 Crisis conformity and affiliation defense: the mortality salience effect

Conformity is a behavior that can be enhanced by death awareness, aligning with the theoretical predictions of mortality salience effects. After being reminded of death, participants are more likely to agree with others when evaluating abstract paintings and commenting on social issues (Renkema et al., 2008; Gao et al., 2020, Study 2). When mortality is salient, donation decisions shift from addressing needs to following others, making social proof appeals more effective than need-based appeals (Cai and Wyer, 2015).

However, key gaps remain in applying existing conformity findings to crisis contexts. First, documented conformity reflects distal defenses (measured post-distraction), managing latent death awareness. During crises, however, decision-making directly addresses immediate life threats, constituting a proximal defense focused on securing actual safety (threat removal) or psychological safety. Research on proximal defenses remains limited, especially regarding conformity as a potential defense. Second, the question of what mechanism enables conformity to buffer mortality threats remains inconclusive. Crucially, the three symbolically grounded distal defenses—cultural worldviews, self-esteem, and close relationships—prove insufficient for explaining crisis-driven conformity when acute mortality threats occupy conscious awareness. This necessitates empirical validation of a proximal defense pathway that mitigates conscious death-related cognition to account for conformity in crisis contexts. As delineated by Mikulincer et al. (2003), TMT categorizes close relationship defenses into two functionally distinct types. One is situational bonding (e.g., reduced interpersonal distance, Wisman and Koole, 2003; light touch, Koole et al., 2014) operating at the preconscious level to deliver immediate anxiety reduction and affective discharge. The other is an enduring connection with significant others, achieving death transcendence through symbolic immortality, a typical distal mechanism. Critically, research confirms that closer physical proximity to the group (even represented by abstract figures) provides a heightened sense of safety (Renkema et al., 2009). Thus, we argue that conformity constitutes a context-dependent, ephemeral affiliation strategy. By fostering intuitive interpersonal alignment, it facilitates proximal psychoregulation during mortality-salient crises.

Affiliation refers to the establishment or maintenance of close interactions with others. Different researchers have similar but not identical conceptual definitions of affiliation, mainly including (1) maintaining a closer physical distance from others (Wisman and Koole, 2003; Renkema et al., 2009); (2) interacting with others, including verbal communication (Marín and Salvador, 2013); (3) establishing further relationships with others (Marín and Salvador, 2013; Jacoby-Senghor et al., 2015); (4) emotionally connecting with others (Hove and Risen, 2009; Marín and Salvador, 2013); and (5) trusting others (O’Connor and Rosenblood, 1996). The evolutionary salience of affiliative bonds is axiomatic: they enhance individual survival and reproductive fitness while buffering against environmental exigencies. The need for affiliation, identified as the desire and behavioral tendency for interpersonal connection, is widespread in humans (Baumeister and Leary, 1995) and is considered one of the three basic needs of individuals (Deci and Ryan, 2000).

Building on the rigorously integrated theorization and empirical evidence presented above, this study argues that affiliation serves as a defense against mortality salience during crises, constituting a core motive for conformity that operates as a proximal defense. This pathway distinctively provides tangible or psychological safety by mitigating conscious mortality anxiety, in contrast to distal defenses that achieve symbolic death transcendence. Rooted in TMT’s affiliation defense mechanisms, conformity may constitute a behavioral manifestation of affiliation-seeking, with its motivational substrate potentially reflecting heightened needs for interpersonal bonding—an underexplored dimension within the TMT paradigm. This study posits that mortality salience induced by crisis contexts activates dual-channel affiliation defenses, where conformity operates as the behavioral stratum and affiliation needs as the motivational stratum, both serving as distinct yet complementary pathways for buffering existential anxiety. Based on TMT’s *mortality salience hypothesis*, we propose Hypothesis 1 as the baseline of this research:

*H1*: Crises increase both affiliation needs and conformity tendencies.

### 1.2 The relationship between crisis-induced conformity and affiliation needs

A critical question arises: What interrelationship exists between conformity and interpersonal affiliation needs as dual manifestations of the defense of interpersonal connectedness? The conceptual boundaries between conformity and affiliation remain overlapping yet distinct. Prior scholarship operationalizes affiliation as physical proximity to groups (Taylor et al., 2000), contrasting with directional conformity, which involves aligning behaviors or opinions with collective norms. Despite behavioral overlaps, critical distinctions emerge. Affiliation does not exclusively target crowds but may focus on specific individuals. Its core function lies in providing psychological security—a primal instinct that operates with minimal reliance on conscious planning or social feedback. Psychobiological research has identified the neurobiological substrates of affiliation. Studies in squirrel monkeys demonstrate that maternal separation elevates cortisol levels, which normalize upon reunion. Human research similarly reveals affiliation’s dependence on precognitive physiological mechanisms (Tronick, 1989; Emde et al., 1991). To clarify this conceptual overlap, we posit that examining behavioral predispositions alongside their underlying motivational drivers constitutes the pivotal lens for both differentiating affiliation from conformity and elucidating their dynamic interplay. Thus, we conceptualize affiliation as an instinct-proximate predisposition that manifests hierarchically affiliative needs (cognitive representation of security-seeking) and conformity propensities (behavioral enactment of collective alignment).

Considering the sequence of psychological processes, affiliative needs may influence conformity tendencies—a relationship supported by some literature and further explored in existing studies. Mikulincer et al. (2003) suggest that individuals’ needs for interpersonal bonds emerge during early developmental stages, prior to the formation of advanced cognitive and decision-making capacities. Baumeister and Tice (1990) posited that affiliative needs drive norm adherence to avoid social exclusion, while Williams et al. (2000) demonstrated that social exclusion (i.e., threatened connection) directly increases conformity. Conformity appears to serve as a behavioral strategy to fulfill the need for belonging. While prior research has established this link in mortality-neutral contexts, the affiliative underpinnings of crisis conformity remain underexplored. Wisman and Koole (2003) found that when participants had to choose between defending their own views (culture worldview defense) and sitting with others (affiliation defense), after being reminded of death, they were more willing to choose to sit with others and even give up insisting on their own views, even though the group was worldview-threatening. Given the unique characteristics of crisis contexts, individuals are hypothesized to exhibit heightened reliance on social support. Building on Hypothesis 1, we next examine how affiliation needs interface with crisis-driven conformity—thereby substantiating that such conformity functions as a death-threat defense mediated by this motivational pathway. Crucially, when mortality threats loom, shifts in affiliation needs would co-vary with conformity intensity. To explore the influence of affiliative needs on crisis conformity, this study posits:

*H2*: Experimental suppression of affiliation needs will lead to a marked reduction in conformity tendencies under crisis conditions.

### 1.3 The buffering effect of crisis conformity based on the anxiety-buffering hypothesis

Guided by TMT’s *anxiety-buffering hypothesis*, crisis conformity’s defensive function can be validated through an alternative lens. Based on the hypothesis, research has found that physical contact with experimenters or plush toys (strengthening interpersonal connection defense) can reduce individuals’ need to seek other defense strategies after being reminded of death (Koole et al., 2014). If crisis conformity is an affiliation defense, then, according to the hypothesis, conformity may alleviate the psychological impact of crisis on individuals. However, despite TMT’s emphasis on defensive coping, few studies have systematically examined whether conformity—especially crisis-specific conformity—serves as an affective defense mechanism to buffer existential anxiety and mortality-related distress. Studies have shown that crises can cause a series of stress-related emotions such as anxiety and fear in individuals (Li et al., 2009). Negative affect triggered by crisis contexts increases uncertainty aversion and cautious crisis decisions, manifested as loss-minimizing (as opposed to gain-seeking) strategies and prolonged decision-making time (Yang and Zheng, 2009). Drawing on TMT’s *anxiety-buffering hypothesis*, crisis conformity may emerge as an adaptive emotion-regulation strategy. Specifically, aligning with others may buffer mortality-salient anxiety (negative affect) while fostering adaptive positivity (e.g., hope, optimism). We hypothesize:

*H3*: In mortality-salient crises, conformity decreases negative affects and increases positive emotions relative to non-conformity, reflecting its anxiety-buffering function.

Synthetically, Hypothesis 1—anchored in TMT’s mortality salience hypothesis—validates that mortality reminders amplify connectedness needs during the proximal defense stage, with conformity serving as their behavioral manifestation (Studies 1a and 1b). Hypothesis 2 further probes the mediational pathway: by manipulating connectedness needs during crises, it tests whether conformity changes covariantly, thereby empirically substantiating its terror management function through this psychosocial conduit (Study 2). Having anchored Hypothesis 1 in TMT’s mortality salience hypothesis to examine the defensive function of crisis-driven conformity and exploring Hypothesis 2 to verify its mediation through affiliation needs (thereby elucidating its affiliation defense mechanisms), Hypothesis 3 shifts focus to TMT’s anxiety-buffering hypothesis, validating conformity’s defensive efficacy through its affective aftereffects—specifically, how crisis conformity modulates mortality-triggered distress (Study 3).

The above hypotheses were tested through four studies. Studies 1a and 1b leverage TMT’s mortality salience paradigm to establish foundational evidence that crisis contexts amplify both affiliation needs and conformity tendencies, forming the baseline for this research. (Study 1b methodologically advances its predecessor by addressing critical limitations in Study 1a’s design). Study 2 then leverages this foundation to investigate the relationship causally. By experimentally manipulating affiliation needs during crises, the covariant adjustment of conformity was tested, thereby validating that crisis conformity operates as a terror management mechanism via this psychosocial pathway. Study 3, shifting to TMT’s anxiety-buffering framework, provides complementary evidence for conformity’s defensive function by mapping its affective aftereffects—specifically, how conformity moderates post-threat distress trajectories.

This study addresses a critical TMT gap: Does crisis conformity serve as an affiliation defense against mortality salience? By testing three hypotheses across four experiments, we present a novel explanation, grounded in affiliation needs and TMT, for why people conform during life-threatening crises.

## 2 Study 1a: the affiliation needs and conformity during crises: testing the mortality salience hypothesis

Study 1 aims to investigate whether crisis contexts (vs. everyday settings) amplify affiliation needs and drive a higher conformity tendency, testing Hypothesis 1: crisis priming increases affiliation motivation as well as conformity tendency, consistent with the mortality salience hypothesis posited by TMT.

### 2.1 Method

#### 2.1.1 Participants and procedure

Using G*Power 3.1, we calculated a required sample size of 102 for an independent-samples *t*-test [one-tailed, *α* = 0.05, power = 0.80, medium effect size *d* = 0.5, as per Kosloff and Greenberg (2009), Burke et al. (2010), Trémolière et al. (2012)]. A total of 139 participants were recruited, with 136 valid responses after excluding those who failed attention checks (*M*_age_ = 18.63 ± 1.15, range = 18–24; 85 females). No participants had prior experience with similar studies. Compensation was provided post-experiment.

Due to ethical considerations, an imaginative scenario task was used to create crisis contexts without exposing participants to any traumatic real-world stimuli. Participants were randomly assigned to either a crisis (*n* = 66) or routine (*n* = 70) condition and completed an online questionnaire. They first participated in a 1-min situational imagination task, followed by sequential measures of need for affiliation and conformity. Demographics and feedback were collected at the end. Debriefing and psychological support were provided after the test.

#### 2.1.2 Materials and measures

##### 2.1.2.1 Situational priming

Participants read a scenario (e.g., “sudden fire during shopping” for crisis vs. “routine shopping” for control) and completed a 1-min imagination exercise. A fill-in-the-blank recall task was used as a manipulation check to ensure participants understood the scenario.

##### 2.1.2.2 Need for affiliation

Adapted from the State Need to Affiliate Questionnaire (Zawadzki, 2009), six 7-point items assessed affiliation tendencies (e.g., “I want to be alone” vs. “I want to be with others”).

##### 2.1.2.3 Conformity

Four 7-point items (two critical) assessed behavioral tendency (e.g., “How likely are you to follow others’ actions?”).

The content validity of all these materials was confirmed by domain experts prior to testing.

### 2.2 Results

#### 2.2.1 Pilot test and manipulation check

To assess the effectiveness of the situational imagination task in activating crisis scenarios, 97 participants were recruited for a pilot study with a one-way between-subjects design using the situational priming materials before the formal experiment. Three 7-point scale items were developed based on the definition of crisis scenarios (danger, uncertainty, life threat). Results showed that participants in the crisis condition perceived their situation as more dangerous (*M*_crisis_ = 5.24, SD = 1.73; *M*_routine_ = 2.54, SD = 1.60; *t*(95) = 8.00, *p* < 0.001, Cohen’s *d* = 1.64, 95% CI = [2.03, 3.37]), more uncertain (*M*_crisis_ = 5.10, SD = 1.54; *M*_routine_ = 2.92, SD = 1.60; *t*(95) = 6.85, *p* < 0.001, Cohen’s *d* = 1.41, 95% CI = [1.55, 2.82]), and more life-threatening (*M*_crisis_ = 5.49, SD = 1.60; *M*_routine_ = 2.38, SD = 1.50; *t*(95) = 9.91, *p* < 0.001, Cohen’s *d* = 2.03, 95% CI = [2.49, 3.74]). These findings confirmed that the priming materials captured the characteristics of crisis scenarios, thereby validating the effectiveness of the situational imagination task. Additionally, post-experiment recall (136/136 correct) in the formal task confirmed scenario understanding and engagement.

#### 2.2.2 Demographic controls

Linear regression showed no significant effects of gender or age on conformity (*F*(2,135) = 1.69, *p* = 0.189), but there were significant effects on affiliation needs (*F*(2,135) = 6.41, *p* = 0.002; *t*_age_ = −2.35, *p* = 0.021; *t*_gender_ = 2.80, *p* = 0.006).

#### 2.2.3 Affiliation needs and conformity tendency

**Figure 1:**
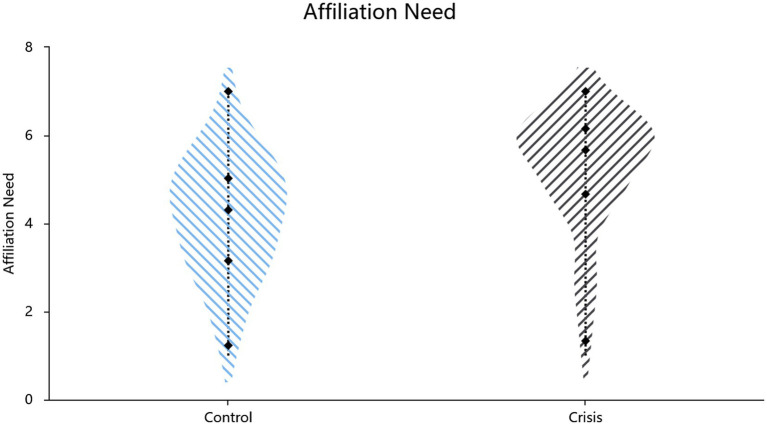
Effect of situational priming on affiliation need.

**Figure 2:**
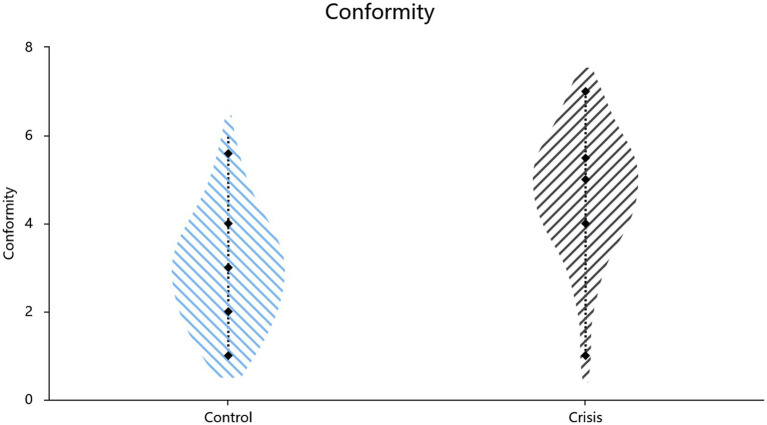
Effect of situational priming on conformity.

Mean scores for affiliation needs (Cronbach’s *α* = 0.84) and the two critical conformity items (Pearson’s *r* = 0.45, *p* < 0.001) served as the corresponding index. Independent-samples t-test results (Table 1; also shown in Figures 1, 2) revealed higher affiliation needs (*t*(134) = 4.63, *p* < 0.001, Cohen’s *d* = 0.80, 95% CI = [0.62, 1.54]) and greater conformity (*t*(134) = 7.96, *p* < 0.001, Cohen’s *d* = 1.38, 95% CI = [1.33, 2.21]) in the crisis condition compared to the routine condition. A hierarchical regression controlling for demographic covariates (gender: *β* = 0.20, *p* = 0.011; age: *β* = −0.16, *p* = 0.050) revealed that the experimental condition (crisis vs. control) significantly predicted increased affiliation needs (*β* = 0.34, SE = 0.23, *p* < 0.001, *R*^2^ = 0.199, *F*[3,132] = 10.96, *p* < 0.001) and conformity (*β* = 0.56, SE = 0.22, *p* < 0.001, *R*^2^ = 0.334, *F*[3,132] = 22.06, *p* < 0.001).

**Table 1:** Effect of situational priming on affiliation needs and conformity.

Situational Priming	Crisis (*n* = 66) Mean (SD)	Control (*n* = 70) Mean (SD)
Need for affiliation	5.24 (1.37)	4.16 (1.34)
Conformity	4.71 (1.36)	2.94 (1.23)

### 2.3 Discussion

Study 1a supported Hypothesis 1: Crisis, as a life-threatening salience, amplifies both affiliation needs and the tendency toward conformity. These findings lay the empirical foundation for integrating TMT’s mortality salience framework into crisis conformity research. Building upon previous theoretical discussions, the heightened affiliation needs and conformity observed after mortality salience likely exemplify the dual-channel affiliation defense, operating simultaneously through motivational drives and behavioral compliance.

In Study 1a, we employed mundane situations as the control condition, following conventional TMT paradigms that contrast mortality salience with neutral daily events (e.g., watching television, Burke et al., 2010). However, the mortality salience effect, wherein death reminders amplify specific psychological tendencies, may stem from the intense negative affect induced by mortality priming (operationalized as crisis contexts in this research) rather than mortality-related cognition per se. To address this limitation, Study 1b employed an enhanced experimental design informed by typical TMT methodology, wherein the control group was exposed to general negative events (e.g., physical pain, academic examinations, uncertainty) rather than affectively neutral scenarios. This refinement enabled us to further isolate and examine the mortality salience effects on affiliation mechanisms encompassing both connection-seeking needs and conformity tendencies under crisis conditions.

## 3 Study 1b: the MS effect of affiliation needs and conformity in life-threatening crisis vs. non-life-threatening crisis

Study 1b employed a refined TMT paradigm (Burke et al., 2010) to rigorously test Hypothesis 1. This methodological refinement involved contrasting mortality salience (operationalized through crisis scenarios) with a control condition featuring general negative events (e.g., pain, worry), as observed in prior research. By employing economic crisis scenarios—a parallel crisis context without direct existential threats—as a control condition, Study 1b enabled granular dissociation of life-threat-specific mechanisms from generalized crisis responses and disentangled the unique effects of mortality from confounding general negative affect. Additionally, measurement protocols incorporated multidimensional specifications of affiliation needs and conformity, enhancing psychometric robustness.

### 3.1 Method

#### 3.1.1 Participants and procedure

Using G*Power 3.1 (with the same parameters as Study 1), 102 valid participants were required. A total of 110 participants were recruited, resulting in 107 valid responses after excluding those with failed post-experiment recall (*M*_age_ = 25.93 ± 6.21, range = 18–56; 64 females). No prior exposure to similar studies; participants were compensated after the experiment.

Participants were randomized into two groups: mortality salience (life crisis, *n* = 52) and control (non-life crisis, *n* = 55) groups, as determined by an online questionnaire. After providing consent, they completed a ≥ 1-min scenario-reading task with manipulation checks, followed by sequential measures of affiliation needs and conformity. Demographics and feedback concluded the survey.

#### 3.1.2 Materials and measures

##### 3.1.2.1 Mortality salience manipulation

The mortality group read a description of the 2008 Wenchuan earthquake, which included casualty data highlighted in red: “69,227 deaths, 374,643 injuries.” The control group read a description of the 2008 financial crisis, highlighting its economic impacts in red: “global GDP contraction of 2.1%.” Four 7-point scales (measuring perceived crisis severity, danger level, likelihood of life threat, and likelihood of property threat) were used as manipulation checks.

##### 3.1.2.2 Affiliation needs (expanded from exp 1)

11 items now include two dimensions of “trust in others” (e.g., “I trust strangers will help in this situation”) and “emotional bonding” (e.g., “I feel connected to others’ emotions”), alongside the original items in Study 1a mainly addressing proximity and interaction.

##### 3.1.2.3 Conformity (expanded from exp 1)

Four items differentiating opinion (e.g., “I change my view to match others”) and behavioral conformity (e.g., “I observe others’ actions to stay consistent”), rated on 7-point scales.

### 3.2 Results

#### 3.2.1 Manipulation check

Independent-samples t-tests (Table 2) revealed no differences between groups in perceived crisis severity (*t*(105) = 1.36, *p* = 0.178, 95% CI = [−0.12, 0.66]) or property threat (*t*(105) = −0.31, *p* = 0.755, 95% CI = [−0.40, 0.29]). The earthquake group reported significantly higher levels of danger (*t*(105) = 3.34, *p* = 0.001, Cohen’s *d* = 0.65, 95% CI = [0.25, 0.97]) and life threat (*t*(87.28) = 7.41, *p* < 0.001, *d* = 1.59, 95% CI = [1.28, 2.22]), confirming the effectiveness of the mortality salience manipulation specific to life-threatening crises.

**Table 2:** Effect of situational priming on crisis severity, death awareness (life threat), affiliation need, and conformity.

Situation priming	Earthquake (*n* = 52) Mean (SD)	Control (*n* = 55) Mean (SD)
perceived crisis severity	6.23 (1.10)	5.96 (0.94)
Perceived danger	6.48 (0.92)	5.87 (0.96)
Perceived life threat	6.40 (0.87)	4.65 (1.51)
Perceived property threat	6.33 (1.00)	6.38 (0.81)
Affiliation needs	5.90 (1.04)	4.95 (0.83)
Conformity	5.28 (0.97)	4.49 (0.96)

#### 3.2.2 Demographic variables

Linear regression showed that the models for affiliation needs (*F*(2, 106) = 4.80, *p* = 0.010) and conformity (*F*(2, 106) = 4.50, *p* = 0.013) were both statistically significant. Specifically, gender had no significant impact on either affiliation needs or conformity, while age had a significant effect on both affiliation needs (*t* = 2.99, *p* = 0.003, 95% CI = [0.02, 0.08]) and conformity (*t* = 2.79, *p* = 0.006, 95% CI = [0.01, 0.08]).

#### 3.2.3 Affiliation needs and conformity

**Figure 3:**
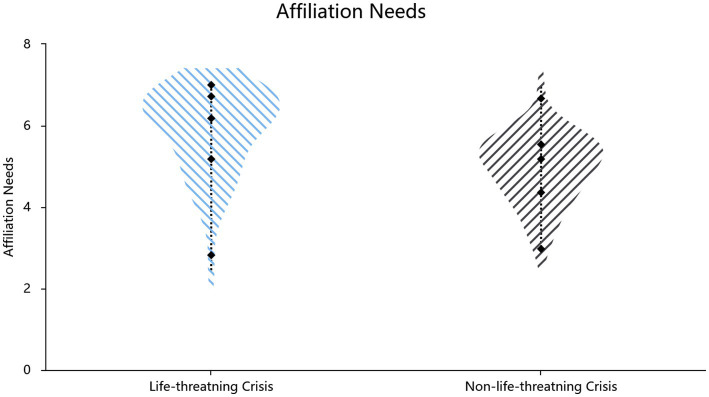
Effect of situational priming on affiliation need.

**Figure 4:**
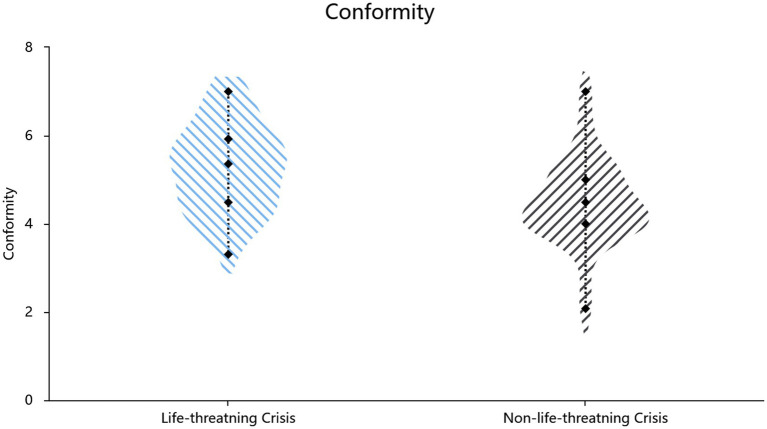
Effect of situational priming on conformity.

Mortality salience increased affiliation needs (*t*(105) = 5.25, *p* < 0.001, *d* = 1.02, 95% CI[0.59, 1.31]; Cronbach’s *α* = 0.93) and conformity (*t*(105) = 4.21, *p* < 0.001, *d* = 0.82, 95% CI = [0.42, 1.16]; Cronbach’s α = 0.84) (Table 2, also shown in Figures 3, 4). Hierarchical regressions controlling for demographic covariates revealed that the experimental condition (crisis vs. control) significantly predicted increased affiliative needs (*β* = 0.43, SE = 0.18, *p* < 0.001, *R*^2^ = 0.270, *F*[3,103] = 12.72, *p* < 0.001) and conformity (*β* = 0.35, SE = 0.18, *p* < 0.001, *R*^2^ = 0.202, *F*[3,103] = 8.68, *p* < 0.001).

### 3.3 Discussion

Study 1b employed the classic mortality salience paradigm of TMT to validate the psychological defense functions of affiliation needs and conformity. The results demonstrated that, compared with death-unrelated crises (i.e., the financial crisis), which are also negative events, in death-related crises (mortality salience), individuals exhibited higher conformity tendencies and affiliation needs, further validating the conclusions from Study 1a, which is consistent with the mortality salience hypothesis in TMT.

In Study 1b, we expanded the measurement of affiliation needs beyond the focus on proximity and interaction in Study 1a by adding dimensions of interpersonal trust and emotional bonding, which are critically relevant to crisis-driven, immediate bonding needs, thereby strengthening the robustness of the measurement. Notably, we excluded the dimension “establishing further relationships,” a core aspect of distal close relationship defenses that has limited relevance during a crisis. Similarly, conformity measurement was refined to distinguish between attitudinal compliance and behavioral synchrony, with internal consistency metrics proving acceptable and the primary findings remaining consistent.

## 4 Study 2: the relationship of affiliation needs and conformity during crises

As distinct manifestations of affiliation at the demand (affiliation needs) and behavioral (conformity actions) level, a critical question arises: How do affiliation needs and conformity interact under crisis contexts? From a sequential psychological processing perspective, affiliation needs may drive conformity behaviors. Prior research has documented that individuals exhibit heightened conformity either to preempt threats to social bonds (proactive regulation; see Baumeister and Tice, 1990) or to compensate for compromised interpersonal connections (reactive compensation; see Williams et al., 2000). To test Hypothesis 2, Study 2 employs experimental manipulation to investigate whether attenuation of affiliation needs causally reduces conformity tendencies under crisis scenarios. Specifically, we predict that manipulated suppression of affiliation needs will significantly attenuate individuals’ propensity for conformity in mortality-salient crisis scenarios.

### 4.1 Method

#### 4.1.1 Participants and procedure

A single-factor (affiliation needs: high vs. low) within-subjects design was conducted, with conformity tendencies serving as the dependent variable. Using G*Power 3.1, we calculated a sample size of 27 for a matched pairs *t*-test [one-tailed, *α* = 0.05, power = 0.80, medium effect size *d* = 0.5, as in Kosloff and Greenberg (2009), Burke et al. (2010), Trémolière et al. (2012)]. A total of 40 participants (*M*_age_ = 23.83 ± 2.79, range 18–23; 33 females) were recruited, all of whom passed manipulation checks and completed the full protocol. None had prior exposure to similar studies; compensation was provided post-experiment.

Upon arrival, participants first completed a crisis-priming scenario imagination task with a written response, followed by the experimental task, which was presented on separate pages. Using a within-subjects design with counterbalanced order, conformity tendencies were assessed under both high and low affiliation need conditions. Afterwards, they completed the manipulation check of the situation priming (free recall of experimental content), provided demographic information, and gave feedback on the experiment.

#### 4.1.2 Materials and measures

##### 4.1.2.1 Situational priming

Similar to Study 1a, participants read a scenario (e.g., “sudden fire during shopping” for crisis) and completed a 1-min imagination exercise. To establish a foundation for subsequent affiliation needs manipulation, the crisis scenario was augmented with the following critical narrative: “*…you notice crowds rapidly evacuating in coordinated groups to find emergency exits, while you remain alone at the scene, spatially isolated from any social clusters.*” A fill-in-the-blank recall task was administered immediately afterward to ensure scenario comprehension as a manipulation check.

##### 4.1.2.2 Need for affiliation

Following the priming phase, participants were exposed to two distinct scenario descriptions. In the *high condition*, participants read: *“You are alone, with no one nearby to discuss with. You must figure out a way to escape the mall quickly by yourself…”* while in the *low need condition* read: *“There happens to be someone nearby. You can discuss with him/her and work together to find a way to escape the mall quickly…”*After reading each scenario, they completed a fill-in-the-blank task targeting key terminology and subsequently made conformity behavioral choices within the contextually framed decision-making paradigm.

##### 4.1.2.3 Conformity

Two 7-point items adapted from Study 1a’s validated paradigm assessed conformity tendency through several behavioral choice questions: *“If you were alone/could partner with someone, how would you prioritize these evacuation options?* (*Follow the crowd = 1, Seek alternative exits independently = 7*)*” and “If you were alone/had a partner and had initially chosen an evacuation route but observed others moving in a different direction, how likely* (*would you be to alter your course to conform with the majority? Definitely not = 1, Definitely will = 7*)*.”*

The content validity of all these materials was confirmed by domain experts prior to testing.

### 4.2 Results

#### 4.2.1 Pilot test and manipulation check

To test the effectiveness of the situational imagination task in manipulating affiliation need, 27 participants (with 22 valid responses) were recruited for a pilot study with a one-way within-subjects design on the situational priming materials before the formal experiment. A 7-point scale was developed to assess perceived affiliation (*“I feel connected to the surrounding crowd”*), and three items were created to assess the need for affiliation (e.g., *“I strongly desire to establish connections with others in the crowd”*) in both scenarios. Additionally, in the companion-present condition, participants completed one item assessing their perceived affiliation (“*I feel connected to this person”*) and three items evaluating affiliation needs toward *the specific companion* (e.g., *“I strongly desire to establish a bond with this person”*).

Paired-samples t-tests revealed significant condition effects. Participants reported lower perceived affiliation toward the crowd in the solitary condition compared to the companion-present condition (*t*(21) = −2.50, *p* = 0.021, Cohen’s *d* = −1.09). Conversely, affiliation needs toward the crowd (Cronbach’s *α* = 0.92) were significantly higher in the solitary condition than in the companion-present condition (Cronbach’s α = 0.89; *t*(21) = 2.44, *p* = 0.024, *d* = 1.06). Furthermore, within the companion-present condition, participants demonstrated weaker connectedness (*t*(21) = −2.74, *p* = 0.012, *d* = −1.19) and lower affiliation needs (*α* = 0.95; *t*(21) = −3.12, *p* = 0.005, *d* = −1.36) toward the crowd relative to their specific companion. Results (Table 3) showed that individuals in solitary scenarios reported weaker crowd connectedness but stronger affiliation needs compared to those with companions, confirming that our manipulation effectively primes high and low affiliation needs. Furthermore, when accompanied during evacuation, individuals exhibited significantly stronger affiliation needs and higher perceived connectedness toward the specific companion compared to the crowd. This pattern aligns with theoretical predictions, indicating that affiliation needs amplified in crisis contexts can be redirected from collective groups to specific individuals.

**Table 3:** Effect of manipulated affiliation on perceived affiliation and affiliation needs in pilot study.

Perceived affiliation/affiliation need	Affiliation target	Scenarios M (SD)	
		Solitary	Companion-present
Perceived affiliation	Toward crowd	3.55 (2.04)	4.59 (1.59)
	Toward companion		5.41 (1.65)
Affiliation needs	Toward crowd	5.33 (1.66)	4.82 (1.70)
	Toward companion		5.59 (1.69)

Additionally, post-experiment recall (40/40 correct) in a formal study confirmed the participants’ understanding and engagement with the scenario.

#### 4.2.2 Conformity

Inter-item correlations between the two conformity items revealed divergence across conditions: they were significant in the high-need scenario (*r* = 0.56, *p* < 0.001) but non-significant in the low-need context (*r* = −0.04, *p* = 0.817), necessitating distinct operationalization as *baseline conformity* (initial compliance) versus *conformity shift* (majority-aligned adjustment).

**Figure 5:**
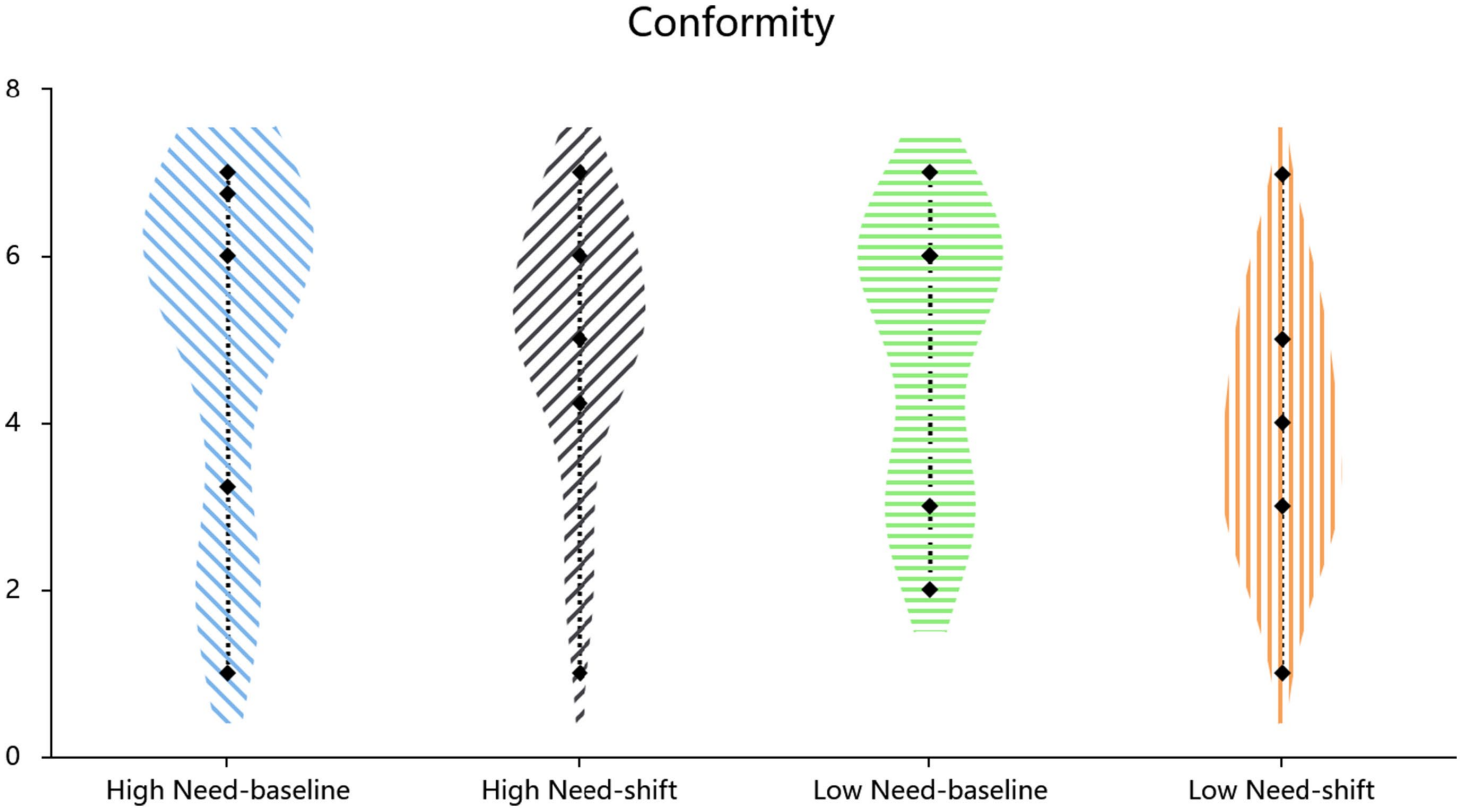
Effect of manipulated affiliation on conformity baseline and shift.

Paired t-tests showed no condition difference in baseline propensity (*t*(39) = 0.60, *p* = 0.555), but there was a significantly lower shift propensity in low- versus high-need conditions (*t*(39) = 5.02, *p* < 0.001, *d* = 0.79) (Table 4; Figure 5).

**Table 4:** Effect of manipulated affiliation on conformity.

Affiliation need	Conformity M (SD)	
	Baseline	Shift
High	5.08 (1.99)	5.05(1.52)
Low	4.88 (1.64)	3.92(1.40)

A 2 × 2 repeated-measures ANOVA revealed a significant main effect of affiliation needs (*F*(1,39) = 9.05, *p* = 0.005, *η*^2^ = 0.19; high: *M* = 5.06, SE = 0.25 vs. low: *M* = 4.40, SE = 0.17). There was a non-significant main effect of conformity type (*F* = 3.76, *p* = 0.060), and a significant interaction (*F* = 6.46, *p* = 0.015, *η*^2^ = 0.14). Simple effects confirmed that (1) there was no baseline-shift difference under high need (*F* = 0.01, *p* = 0.926); (2) shift propensity was reduced compared to baseline conformity under low need (*F* = 7.49, *p* = 0.009, *η*^2^ = 0.16); and (3) there was a significant reduction in shift propensity as affiliation needs toward the crowd decreased (*F* = 25.19, *p* < 0.001, *η*^2^ = 0.39), but at not baseline (*F* = 0.35, *p* = 0.555), which is consistent with the t-test results.

### 4.3 Discussion

Study 2 examined how manipulated affiliation needs (high: solitary evacuation vs. low: companion-coordinated evacuation) influence conformity. Results showed that participants consistently prioritized initial alignment with the crowd across both conditions, potentially reflecting a crisis-induced affiliative drive to merge the self into a larger collective. Critically, when affiliation needs were reduced via companion presence (pretest-confirmed reduced crowd affiliation needs), the tendency to shift from initial decisions toward conformity decreased significantly compared to high-need contexts. This verified Hypothesis 2, together with a significant main effect of affiliation needs in the 2 × 2 repeated-measures ANOVA. These findings demonstrate that while crisis contexts universally prompt baseline conformity, affiliation needs significantly affect subsequent conformity adjustments. This dissociation indicates that interpersonal bonds can selectively target individuals rather than groups—when dyadic affiliation needs are met, crowd-directed affiliation diminishes accordingly.

## 5 Study 3: defensive efficacy of crisis conformity: testing the anxiety-buffering hypothesis

Guided by the affective regulation framework of defense mechanisms (Hart, 2014), Study 3 employed a 2 (conformity decision: alignment vs. independence) × 2 (context: mortality-salient crisis vs. mundane routine) factorial design to investigate context-dependent emotional modulation through affiliation strategies (i.e., conformity). Based on the *anxiety-buffering hypothesis* in TMT, Study 3 verifies the defensive function from the perspective of the emotional aftermath of crisis conformity, testing Hypothesis 3, which posits that crisis conformity effectively reduces individuals’ negative affects and increases positive emotions.

### 5.1 Method

#### 5.1.1 Participants and procedure

A 2 (situation: crisis/daily) × 2 (conformity/non-conformity) two-factor mixed design was conducted. Using G*Power 3.1, we calculated a sample size of 98 for a mixed-design ANOVA (*f* = 0.25, referring to Kosloff and Greenberg (2009), Burke et al. (2010), Trémolière et al. (2012); *α* = 0.05 [one-tailed], power = 0.80). A total of 102 participants (*M*_age_ = 25.30 ± 7.42, range 18–58; 73 females) were recruited, all of whom passed manipulation checks and completed the full protocol. Participants had no prior exposure to similar studies, and compensation was provided post-experiment.

Upon arrival, participants completed 1–2 min of guided breathing to calm down. Then, they were randomized into either the crisis (*n* = 52) or routine (*n* = 50) groups. In the formal part of the experiment, participants first carried out a situation imagination and writing task. Next, they completed emotion assessments across two decision contexts (conformity vs. non-conformity) presented in a counterbalanced order via separate experimental modules. Afterward, they completed a manipulation check of the situation priming, provided demographic information, and gave feedback on the experiment.

#### 5.1.2 Materials and measures

##### 5.1.2.1 Situation priming

Similar to Study 1, participants completed a 1-min imagination and writing task about a sudden disaster where they are, such as shopping in a mall. As a manipulation check, they rated how dangerous, life-threatening, and uncertain they viewed the crisis or routine situation to be.

##### 5.1.2.2 Conformity manipulation

For the crisis group, they read scenario descriptions in the conformity situation as *“You see everyone around you running in a certain direction, and you choose to run in the same direction as them”* while in the non-conformity situation as *“You see everyone around you running in a certain direction, and you choose to run alone in a different direction.”* For the routine group, the conformity situation was *“Choosing the same product as those around you,”* and the non-conformity situation was *“Going to another place alone and choosing a different product.”*

##### 5.1.2.3 Affects

In both conformity and non-conformity situations, emotion ratings included eight negative (such as anxiety, fear, etc.) and five positive emotions (such as calmness, optimism), validated by 10 experts (content validity confirmed). The crisis group additionally rated “death worry” in both conformity and non-conformity scenarios.

### 5.2 Results

#### 5.2.1 Manipulation check

Results of independent samples t-tests showed that participants in the crisis group rated their situation as significantly more dangerous (*M*_crisis_ = 5.35, SD = 1.51, *M*_control_ = 2.22, SD = 1.22, *t*(100) = 11.50, *p* < 0.001, Cohen’s *d* = 2.30, 95% CI = [2.65, 3.59]), more uncertain (*M*_crisis_ = 5.33, SD = 1.46, *M*_control_ = 2.68, SD = 1.35, *t*(100) = 9.49, *p* < 0.001, Cohen’s *d* = 1.90, 95% CI = [2.20, 3.19]), and more life-threatening (*M*_crisis_ = 5.62, SD = 1.32, *M*_control_ = 1.98, SD = 1.20, *t*(100) = 14.54, *p* < 0.001, Cohen’s *d* = 2.91, 95% CI = [3.17, 4.03]) than the routine group, confirming the manipulation of the crisis.

#### 5.2.2 Negative emotions

**Figure 6:**
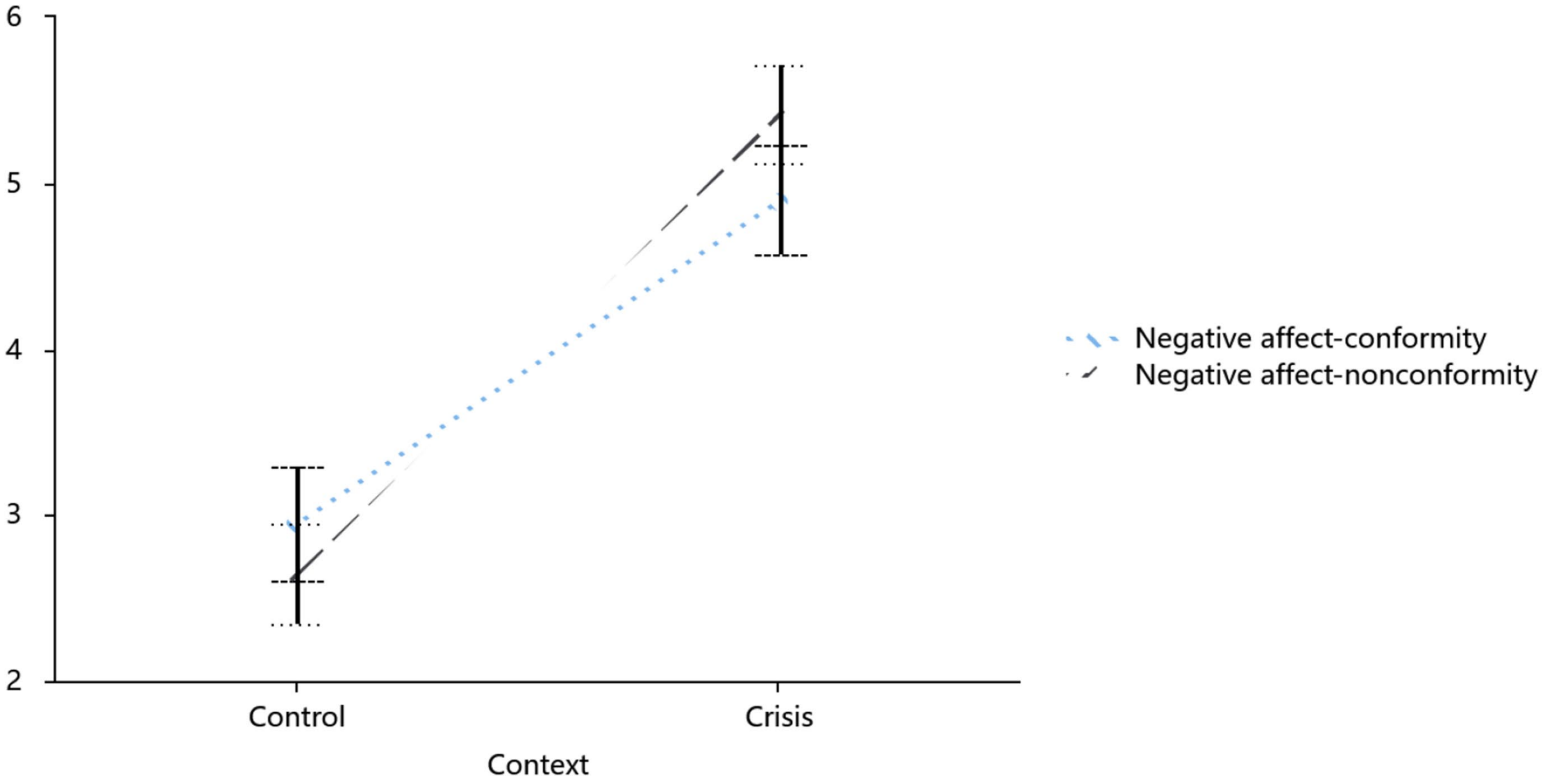
Influence of crisis and conformity on negative affect.

Negative emotion scores (*α* = 0.96 for both conformity and non-conformity) revealed a significant 2 × 2 interaction (*F*(1,100) = 12.39, *p* = 0.001, *η*^2^ = 0.11): crisis participants reported lower negative affect in conformity (*M* = 4.78, SD = 1.47) than in non-conformity (*M* = 5.44, SD = 1.29; *F*(1,100) = 10.04, *p* = 0.002, 95% CI = [−0.86, −0.17]). In contrast, routine participants showed no such benefit (3.13 vs. 2.74, *p* = 0.071). This interaction was significant, with a strong main effect of situation (*F*(1,100) = 93.64, *p* < 0.001, *η*^2^ = 0.48), where crisis contexts overall elicited higher negative emotions than routine contexts (see Figure 6).

In addition, crisis participants (*n* = 52) reported lower death worry when conforming (*M* = 4.62, SD = 1.71) compared to when not conforming (*M* = 5.42, SD = 1.55; paired *t*(51) = −3.54, *p* = 0.001, 95% CI [−1.11, −0.32]), directly aligning with TMT’s anxiety-buffering prediction.

#### 5.2.3 Positive emotions

**Figure 7:**
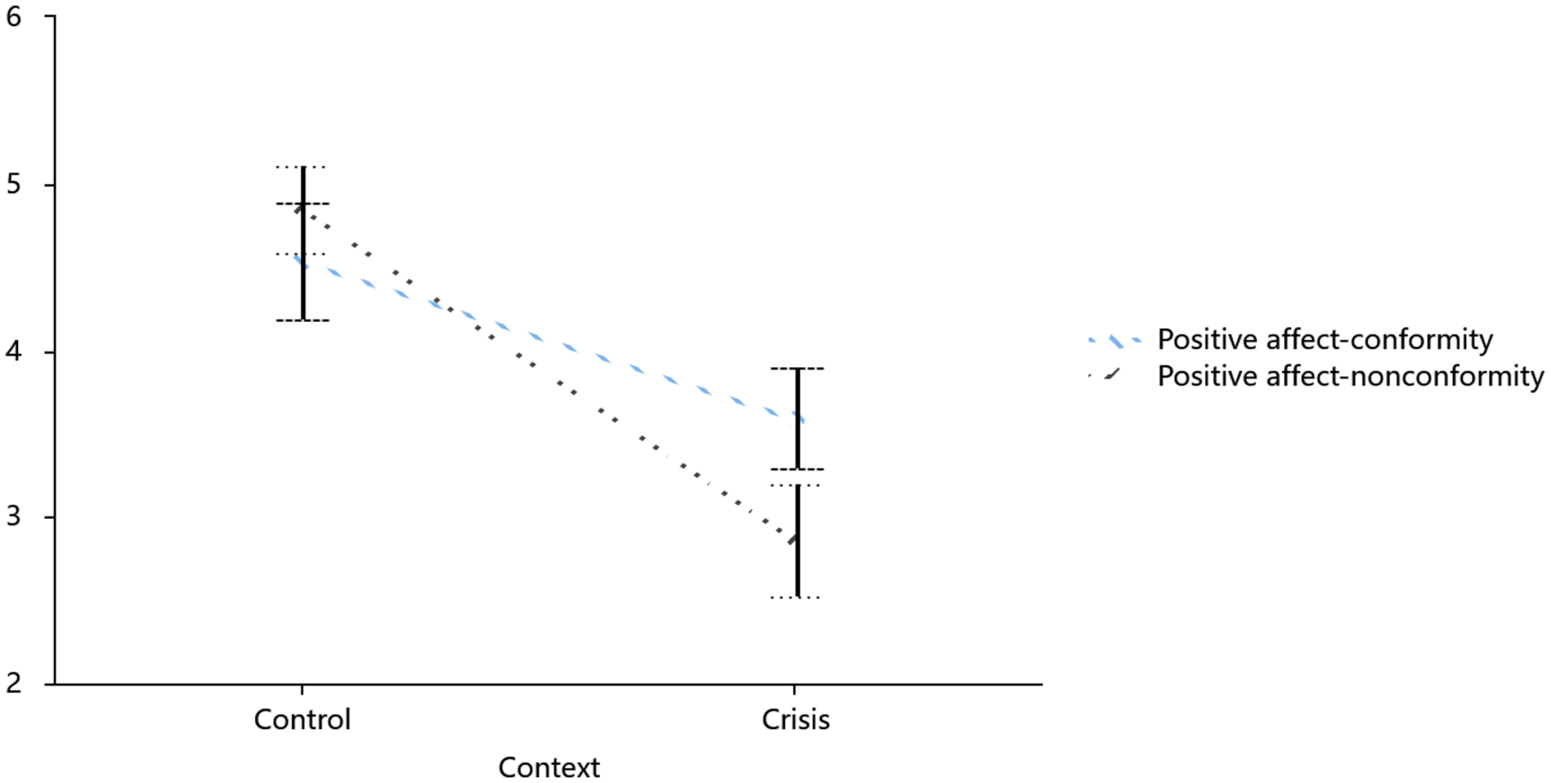
Influence of crisis and conformity on positive affect.

Similar to the analysis of negative emotions, positive emotions (*α* = 0.86/0.88 for conformity/non-conformity) showed a significant situation × conformity interaction (*F*(1,100) = 15.55, *p* < 0.001, *η*^2^ = 0.14): crisis participants reported higher positivity in conformity (3.67 vs. non-conformity 2.88; *F* = 13.92, *p* < 0.001, 95% CI = [0.38, 1.08]), while routine participants showed the slightly opposite trend (4.27 vs. 4.67, *p* = 0.065). This occurred alongside a strong main effect of situation (*F* = 33.92, *p* ≤ 0.001, *η*^2^ = 0.25), with crises reducing overall positive affect compared to routines (Figure 7).

#### 5.2.4 Differences in covariation between crisis situational characteristics and emotion under conformity vs. non-conformity

To compare the predictive effects of danger on negative emotions between conformity (*r* = 0.535) and non-conformity (*r* = 0.761) contexts, a Steiger’s *Z*-test was conducted to evaluate the difference in correlation strength. The results demonstrated a statistically significant divergence (*Z* = −4.76, *p* < 0.001, Δ*r* = 0.226, 95% CI [0.14, 0.31]). Similar patterns were also observed in the predictive effects of uncertainty (*Z* = −2.62, *p* = 0.009, Δ*r* = 0.129, 95% CI [0.04, 0.22]) and life-threatening situations (*Z* = −6.40, *p* < 0.001, Δ*r* = 0.256, 95% CI [0.18, 0.33]) on emotional responses, suggesting cross-domain consistency in crisis perception mechanisms. Furthermore, similar effects were observed in the predictive effects of danger, uncertainty, and life-threatening situations on positive emotion.

This indicates that in non-conformity crisis scenarios, the covariation between emotions and situational risk features was more pronounced than in conformity contexts, partially suggesting a buffering effect of conformity on emotional responses to environmental threats.

### 5.3 Discussion

Study 3 revealed that in crisis contexts, conformity significantly reduced negative affects (e.g., anxiety, fear, uncertainty) and death worry while increasing positive emotions (e.g., positivity, optimism, security), compared with nonconformity. No such effects emerged in routine situations. This confirms that crisis conformity serves as a psychological defense mechanism, aligning with TMT’s anxiety-buffering hypothesis (Hypothesis 3)—conformity mitigates mortality-salience-induced distress and fosters positivity during crises. The divergent predictive patterns of crisis characteristics across conformity and non-conformity contexts provided empirical support for this conclusion, highlighting the regulatory role of social conformity in crisis adaptation.

## 6 General discussion

This research uncovered crisis conformity as a mortality-salience-triggered affiliation defense, as tested in Hypotheses 1–3, empirically linking it to anxiety reduction and providing a novel TMT perspective on its adaptive mechanism.

### 6.1 The psychological defense function and adaptability of crisis conformity

Conformity is ubiquitous, yet existing frameworks—rational theories (which optimize decisions via full information, Raafat et al., 2009) and heuristic theories (which are ecological shortcuts for bounded rationality, Kahneman and Tversky, 1979)—fall short in explaining crisis contexts. Rational theories fail because the majority of choices during crises rarely reflect information sufficiency (e.g., fleeing blindly during earthquakes), and heuristic theories lack mechanistic clarity on why threat triggers conformity shortcuts.

This study addresses this gap by uncovering crisis conformity as a mortality-salience-driven psychological defense: Although not objectively optimal, it buffers existential distress (negative affect and death worry in Study 3) and enhances affiliative security, enabling adaptive action (Li et al., 2009; Yang and Zheng, 2009).

From a TMT affiliation defense perspective, crisis conformity complements rational and heuristic theories: rational utility expands to include “emotional utility” (e.g., reduction in mortality anxiety, Study 3), while heuristic triggers gain depth—mortality salience-induced affiliation needs and conformity (Studies 1–2). This extends ecological rationality (Kruglanski and Gigerenzer, 2011) to include existential adaptivity—conformity serves not just as a cognitive shortcut but as a deeply ingrained psychological mechanism for buffering mortality anxiety (Li et al., 2009), even when objective safety remains uncertain.

### 6.2 Crisis conformity: new insights into terror management’s proximal relational defenses

Terror Management Theory posits that awareness of mortality typically activates distal defenses—delayed, symbolic strategies measured after distraction tasks, such as worldview defense (Burke et al., 2010). Affiliation defenses in prior TMT research, like increased partner longing (Mikulincer and Florian, 2000) or proximity-seeking (Wisman and Koole, 2003), emerged after mortality salience priming with distraction intervals, reflecting distal processes.

This study introduces a novel approach by measuring affiliation needs and conformity immediately after crisis priming, within the same threat context, with no temporal delay. This design captures mortality awareness still at the proximal defense stage (conscious awareness), demonstrating that affiliation defenses (e.g., conformity) can operate proximally—a novel finding. While proximal affiliation defenses are underexplored, our results align with Lu et al.’s (2019) priority hypothesis: when death is salient, individuals prioritize relational defenses before distal strategies. The immediate defensive effect of affiliation conformity in our study, which reduces death worry (Study 3), likely reflects this prioritization, filling a critical gap in TMT’s proximal defense literature.

Previous studies have raised concerns regarding the robustness of the mortality salience effect (Klein et al., 2019). However, TMT scholars Chatard et al. (2020) argue that the initial analysis by Klein et al. (2019) reflects a Type II error risk stemming from methodological flaws, rather than the inherent unreliability of the effect itself. Through methodological refinements (strict procedural protocols, sample size control, and cultural calibration), they demonstrate that the mortality salience effect retains robustness when adhering to TMT’s core theoretical paradigms. The ongoing controversy underscores the necessity for refined replication studies to clarify boundary conditions, rather than invalidating the theoretical framework as a whole. Notably, the mortality salience effect questioned by Klein et al. (2019) pertains to distal defenses, where dependent variables are assessed after delay and primarily involve tasks with no direct relevance to death-related contexts.

In contrast, this study focuses on proximal defenses, which are theoretically predicted to exhibit stronger effects than distal ones. This distinction highlights the need to distinguish between proximal and distal defense mechanisms. Crucially, empirical investigations of proximal defenses within TMT remain substantially underrepresented compared to those of distal defenses. Our research directly addresses this critical gap in the literature.

### 6.3 Relationship between affiliative needs and conformity propensity in crisis contexts

In this study, we propose that affiliation needs and conformity represent two levels of affiliation defense mechanisms activated by crisis contexts, such as mortality salience. Affiliation, as a primal motivational drive that precedes cognitive processing and behavior, has been theoretically constructed and empirically validated as part of a close relationship defense mechanism against mortality salience. Existing studies have explored various manifestations of affiliation across cognitive and behavioral domains (e.g., prosocial cognitions, physical proximity-seeking behaviors, Mikulincer et al., 2003), which serve as concrete observable indicators of affiliation processes.

Specifically, in our framework, affiliative needs reflect the cognitive stratum (the subjective craving for social bonds), and conformity propensity embodies the behavioral stratum (the intentional alignment with collective actions). Prior research demonstrates that individuals may abandon their personal opinions to maintain spatial closeness with others after mortality salience is invoked (Renkema et al., 2008). Studies 1a and 1b provide evidence that both affiliation needs and conformity propensity satisfy the mortality salience hypothesis, functioning as parallel defensive responses.

Building on these results, we further investigated how affiliative needs and conformity dynamically interact. Study 2 preliminarily revealed that (1) individuals tend to exhibit heightened conformity during crises, preferring collective integration that merges the vulnerable self into larger groups, regardless of companion presence. (2) Fluid affiliative compensation: While crisis contexts amplify affiliative needs, these needs can be flexibly channeled toward either crowds or specific individuals (e.g., evacuation companions). (3) Substitution effect: When affiliative bonds with specific individuals partially satisfy crowd-directed needs, both affiliative cravings toward crowds and conformity propensities significantly diminish. These intriguing findings align with the existing literature on affiliation-conformity linkages (Baumeister and Tice, 1990; Williams et al., 2000), collectively suggesting that crisis conformity may serve as a strategy to fulfill affiliation needs and cultivate relational security, at least in part through dyadic bonds.

### 6.4 Contributions and limitations

Theoretically, this study introduces affiliation as a vital underlying mechanism of crisis conformity, extending prior studies (Williams et al., 2000; Renkema et al., 2009) to life-threatening contexts. For the first time, it integrates crisis conformity into Terror Management Theory, empirically validating its affiliation defense function—buffering mortality anxiety and negative affect and filling TMT’s proximal defense gap.

In practice, this study offers novel insights for public crisis management. During crises, promoting affiliative behaviors, such as family connections, mutual assistance, and volunteering, can help curb irrational conformity, as supported by Study 2. Leveraging the need for affiliation and crisis-related mortality salience can nudge essential actions, such as pandemic mask-wearing, by steering individuals toward scientific conformity. Moreover, enhancing awareness of crisis-induced affiliation needs enables individuals to better manage distress.

This study has some limitations. Methodologically, ecological validity could be enhanced. We adopted field-validated laboratory methods, which are standard in crisis research due to ethical and feasibility considerations (Rottenberg et al., 2007; Xing et al., 2015). While ecological validity is limited, our manipulation checks (recall accuracy) and previous evidence of laboratory and real-world emotion equivalence support the internal validity of our findings. Future work could employ immersive technology (e.g., VR) and multimodal measures (physiology and behavior) for richer crisis simulations.

Besides, three critical gaps merit future exploration. First, while crisis conformity’s affiliation defense operates acutely (proximal stage), does it persist in TMT’s delayed distal stage (e.g., after a 20-min distraction)? Conformity tendency and need for affiliation in the distal defense phase remain to be examined. Second, while Study 2 confirms the affiliation defense mechanism of crisis conformity, does affiliation needs to mediate crisis-driven conformity in causal inference terms? Future research requires larger-sample mediation analyses to statistically validate this causal pathway. Third, do other intrinsic motivations drive conformity? Future research could leverage TMT’s worldview and self-esteem defense frameworks to disentangle the motivational heterogeneity of conformity, aligning with TMT’s triadic model. Finally, while crisis conformity buffers emotions, how does it affect cognitive functions? Future studies might investigate its role in threat-related memory (e.g., flashbulb memories) or decision-making biases, uncovering adaptive and maladaptive trade-offs.

Beyond mortality salience effects (TMT), shifts in affiliation needs and conformity during crises may be alternatively explained by epistemic theories (e.g., uncertainty management, meaning maintenance). The decades-long theoretical debate between TMT and other psychological defense frameworks persists (Hart, 2014). Crucially, empirical evidence confirms that self-uncertainty mediates the link between mortality salience and conformity, exploring interdependencies among explanatory mechanisms underlying defensive processes (Gao et al., 2020). We contend that explanatory pluralism is necessary: human motivation during crises is multidimensional, resisting reduction to a single theoretical lens. A shared defensive logic unites these theories: whether via TMT, uncertainty reduction, or meaning maintenance, the core drive is to alleviate psychological threats. Theoretically, conceding that critical unknowns remain, future research must disentangle whether these threats are distinct constructs or manifestations of a latent defensive system, and how their interactions shape social bonding and conformity.

## 7 Conclusion

This study reveals the psychological mechanism of crisis conformity within the framework of TMT: crises increase affiliation needs and conformity (Studies 1a & 1b), which may represent dual-strata manifestations of affiliation defense within TMT. Furthermore, affiliative needs influence conformity in crisis contexts: when affiliative bonds with specific individuals partially satisfy crowd-directed needs, both affiliative cravings toward crowds and conformity propensities significantly decrease (Study 2). Conformity, as an affiliation defense, serves to mitigate negative affect and mortality anxiety while enhancing positive affect, thereby buffering psychological distress (Study 3).

## Data Availability

The original contributions presented in the study are included in the article/supplementary material, further inquiries can be directed to the corresponding author.
